# Breast cancer leptomeningeal metastasis: propensity of breast cancer subtypes for leptomeninges and the analysis of factors influencing survival

**DOI:** 10.1007/s12032-012-0408-4

**Published:** 2013-01-16

**Authors:** Anna Niwińska, Halina Rudnicka, Magdalena Murawska

**Affiliations:** 1 Department of Breast Cancer and Reconstructive Surgery, The Maria Skłodowska-Curie Memorial Cancer Center and Institute of Oncology, 5 Roentgen str., 02-781 Warsaw, Poland; 2 Department of Biostatistics, Erasmus University Medical Center, Rotterdam, The Netherlands

**Keywords:** Breast cancer biological subtype, Carcinomatous meningitis, Leptomeningeal metastasis, Lobular cancer, Neoplastic meningitis, Triple-negative breast cancer

## Abstract

The aim of the study was to define biological subtypes of breast cancer that have the propensity to metastasize to the leptomeninges and to assess factors influencing survival from detection of leptomeningeal metastatis (LM). One hundred and eighteen consecutive breast cancer patients with LM were treated in one institution, between the years 1999 and 2009; 40.5 % of patients had triple-negative subtype, 37.5 % had luminal A subtype and 22 % had HER2-positive subtypes (luminal B and HER2). Of patients with LM, 35 % had lobular cancer. Median survival from the detection of LM was 18 weeks, and 1-year survival was 16 %. Cox multivariate analysis revealed that performance status and systemic treatment statistically significantly influenced survival of patients with LM. Triple-negative biological subtype and lobular histological type of breast cancer had the propensity to metastasize to the leptomeninges. Performance status and systemic treatment ordered after detection of LM statistically significantly influenced survival.

## Introduction

Leptomeningeal metastasis (LM) is a deleterious complication of breast cancer leading to death within less than 4–6 months following the diagnosis [1–4]. It touches between 2 and 5 % of patients with metastatic breast cancer, usually later in the course of their disease. LM presents a challenge for an oncologist because of the difficulty in determining the diagnosis and lack of optimal therapy [1, 2, 4]. Early diagnosis of LM is important in order to prevent the development of severe neurological deficits that cannot be reversed with treatment. Usually the treatment requires focal radiotherapy to symptomatic sites or areas of bulky disease followed by intrathecal chemotherapy or systemic intravenous treatment, but there is conflicting data regarding the efficacy of particular type of treatment.

Due to the fact that LM is becoming an increasingly common complication of breast cancer [2, 4] it is important to know which histological and biological type of newly diagnosed breast cancer has the propensity to metastasize to the leptomeninges and what type of treatment of LM is mostly effective.

The first aim of the present study was to define biological subtypes that have propensity to metastasize to the leptomeninges. The second goal was to assess factors influencing survival from detection of LM with a focus on particular treatment methods.

## Materials and methods

Between the years 1999 and 2009, 118 consecutive breast cancer patients had been treated for LM at the Department of Breast Cancer of the Maria Sklodowska-Curie Memorial Cancer Center and Institute of Oncology in Warsaw, Poland. The observation of patients started at the time of the detection of leptomeningeal metastases, and all data were collected prospectively in the database. In each case, treatment options were approved by multidisciplinary team of neurologist (H.R.), radiation oncologist (A.N.) and medical oncologists and were performed after patients had signed written consent form. Clinical characteristics of the entire group are presented in the Table 1.

**Table 1:** Patient’s characteristics (*n* = 118)

Features	Number of patients	Rate (%)
Age at initial diagnosis (median, range, years)	49 (21–74)	
*TNM at initial diagnosis*		
I	7	6
II	41	35
III	50	42
IV	20	17
*Histology of primary tumor*		
Ductal carcinoma	54/92	59
Lobular carcinoma	32/92	35
Other types (medullary, metaplastic)	6/92	6
Cancer cells without type assessment ^a^	26	–
*Estrogene/progesterone receptors*		
Negative	60	51
Positive	49	42
Missing	9	7
*HER2 receptor*		
Negative	77	65
Positive	22	19
Missing	19	16
*KPS*		
≥70	44	37
<70	74	63
*Site of distant metastases*		
Lung	42	36
Liver	29	25
Bones	57	48
Brain (parenchyma)	45	38
Locoregional failure	43	36
Leptomeninges as the only site of metastases	29	25

^a^In 26 patients with locally advanced or disseminated breast cancer, the diagnosis was established by fine needle biopsy before systemic therapy

In order to confirm the diagnosis of LM, patients underwent neurological examination, lumbar puncture with the detection of cancer cells in cerebrospinal fluid (CSF) and magnetic resonance imaging (MRI).

Table 2 shows the treatment methods in details. In 66 patients (56 %) with bulky disease or clinical symptoms, whole brain radiotherapy was performed and in 28 cases (24 %) spinal leptomeninges were irradiated. In 92 patients (78 %), intrathecal methotrexate (10 mg dose) together with dexamethasone (4 mg dose) was given. At the onset of treatment, these drugs were administered twice a week and once a week after clinical improvement was achieved. The intrathecal treatment was maintained until the normalisation of CSF parameters or progression of the disease. Seven courses were administered (range 1–15 doses) on average. In 2 patients, intrathecal liposomal cytarabine was administered. In 80 patients (68 %), systemic chemotherapy was administered and in a majority of them it started after the completion of radiotherapy and/or after the intrathecal treatment. Systemic chemotherapy was ordered in patients with LM and concurrent parenchymal metastases. Programs with vinorelbine, anthracyclines, capecitabine, platinum salts or taxanes were usually administered. Without having the possibility to perform gene expression profiling, biological subtypes of brain metastases were defined based on the expression of oestrogen (ER), progesterone (PgR) and HER2 receptors [5]. Out of 118 patients, 99 were divided into four biological subsets. Nineteen patients were unassigned because of insufficient tumour material for assay. Immunohistochemistry (IHC) staining was performed on tissue sections that were cut from formalin-fixed and paraffin-embedded primary breast tumours. Fluorescence in situ hybridisation (FISH) was used for all HER2 2+ tumours. HER2-positive staining was defined as IHC3+ or in the case of IHC 2+-FISH positive. HER2-negativity was defined as IHC 0, 1+ or 2+ along with negative FISH results. Patients were divided into four biological subtypes: (1) triple-negative (ER-negative, PgR-negative, HER2-negative), (2) HER2 (HER2-positive, ER-negative, PgR-negative), (3) luminal B (HER2-positive, ER-positive and/or PgR-positive) and (4) luminal A (ER-positive and/or PgR-positive HER2-negative). HER2 and luminal B subsets were HER2-positive.

**Table 2:** Type of treatment for LM

Type of treatment	Number of patients	Rate (%)
Intrathecal chemotherapy	93	79
Systemic therapy	80	68
Whole brain radiotherapy	66	56
Spinal cord radiotherapy	28	24
*Type of intrathecal therapy*		
Methotrexate		
(10 mg/dose, median total dose −70 mg)	92	78
Liposomal cytarabine	2	2
*Type of systemic treatment*		
Chemotherapy	75	64
Hormonal therapy	19	16
Targeted therapy	8	7
*Type of chemotherapy* ^*a*^		
Vinorelbine	24	20
Antracyclines	21	18
Capecitabine	15	13
Platinum salts	11	9
Taxanes	10	8
Fluorouracil	9	8
Etoposide	7	6
BCNU	6	5
Cyclophosphamide	6	5
Temozolomide	3	3
*Type of hormonal therapy*		
Aromataze inhibitors	15	13
Tamoxifen	8	7
Gosereline	6	5
*Type of targeted therapy*		
Trastuzumab	7	6
Lapatinib	1	1
*Intensity of CM treatment*		
Three methods of treatment used	30	25
Two methods of treatment used	49	42
One method of treatment used	32	27
No treatment	6	5

^a^Most patients received many types of systemic treatment

### Statistical analysis

Descriptive statistics were used to determine patient demographics and clinical characteristics. Hypothesis tests were conducted at the alpha = 0.05 level with a 95 % confidence interval. In order to compare categorical tumour features in the 4 biological subgroups of patients, the chi square test was used. For those categorical variables in which the chi square test was inappropriate because of small sample size, the Fisher exact test was used. Univariate analysis and Cox proportional hazards model were developed to identify factors influencing survival from LM. The following factors were analysed: age at LM (≤50 vs. >50), Karnofsky performance status (KPS, ≤70 vs. >70), histological type (lobular vs. ductal), biological subtype (triple-negative vs. HER2-positive and triple-negative vs. luminal A), distant metastases/locoregional recurrence (present vs. absent), lung metastases (present vs. absent), liver metastases (present vs. absent), parenchymal brain metastases (present vs. absent), bone metastases (present vs. absent), radiotherapy to the spinal cord (yes vs. no), radiotherapy to the brain (yes vs. no), intrathecal treatment (yes vs. no) and systemic treatment (yes vs. no). Disease-free survival, overall survival, survival from recurrence of the disease to LM and survivals from the detection of LM in the entire group and in the four biological subgroups were estimated using the Kaplan–Meier method and compared using the log-rank test.

## Results

The most common neurological symptom, observed in 54 % of patients was headache, followed by cranial nerves symptoms (42 %), cerebellar signs (35 %), nausea/vomiting (30 %), parhesis (26 %), mental changes (19 %), meningism (11 %), seizures (9 %) and radicular pain (7 %). In 114 out of 118 patients, LM was diagnosed based on the demonstration of cancer cells in CSF. In four patients characteristic enhancement of leptomeninges in MRI, together with neurological signs and symptoms confirmed the diagnosis; in those patients, lumbar puncture was contraindicated because of coexisting parenchymal brain metastases with signs and symptoms of high intracranial pressure. Pre-treatment analysis of the CSF revealed increased cytosis in 76 % of patients, increased protein level in 76 % of patients and decreased glucose level in 63 % of patients. The initial MRI revealed diffused enhancement of leptomeninges in 114 patients (97 %), tumour nodules in 14 patients (12 %), secondary hydrocephalus in 13 patients (11 %) and parenchymal brain metastases which coexisted with LM in 45 patients (38 %). The initial NMR images were normal in 9 patients (8 %). Histological type of breast cancer was accessible in 92 patients (in 26 patients with III and IV clinical stages only fine needle biopsy of the primary tumour was performed with the detection of cancer cells); 35 % of patients had lobular cancer or mixed histology with lobular component, 59 % had ductal cancer and 6 % had other histological types of cancer (medullary, metaplastic, adenoids cysticum) that usually correlate with the triple-negative biological subtype.

Biological subtypes were assessed in 99 patients. Among biological subtypes, triple-negative (40.5 %) and luminal A (37.5 %) were the most commonly presented; 22 % of patients had HER2-positive subtypes (luminal B-8 % and HER2-14 %).

In 29 cases (25 %), LM occurred as an isolated site of relapse, in 89 patients (75 %) distant metastases and/or locoregional failure were discerned. Karnofsky performance status (KPS) ≥70 was assessed in 44 (37 %) and <70 in 74 (63 %) of patients.

The analysis within biological subsets is presented in Table 3. It revealed the differences in histological types distribution and pattern of distant metastases. Ductal carcinoma was almost evenly distributed within four biological subtypes, but lobular cancer was the most frequent in luminal A subtype (52 % of all cases). There was no difference in the distribution of lung metastases within biological subtypes, but liver metastases were more frequent in luminal A and luminal B subtypes. Bone metastases were observed mostly in patients with luminal A, and brain parenchymal metastases were typical for luminal B and HER2 subtypes.

**Table 3:** Patient’s characteristics within biological subtypes (99 patients)

Feature	Triple-negative (ER-negative, PgR-negative, HER2-negative)	HER2 (ER/PgR-negative, HER2-positive)	Luminal B (ER/PgR-positive, HER2-positive)	Luminal A (ER/PgR-positive, HER2-negative)	*p* value
		HER2-positive 22 (22 %)			
Number of patients	40 (40.5 %)	14 (14 %)	8 (8 %)	37 (37.5 %)	
Age at initial diagnosis (years)	49	50	55	47	0.993
Age at LM (years)	51	50	55	50	0.493
Histological type:					
Ductal carcinoma	21/33 (64 %)	6/7 (86 %)	4/6 (67 %)	15/31 (48 %)	
Lobular carcinoma	6/33 (18 %)	1/7 (14 %)	2/6 (33 %)	16/31 (52 %)	
Other types (medullar, metaplastic carcinoma)	6/33 (18 %)	0	0	0	
Cancer cells without type^a^	6	7	2	6	0.006
Karnofsky performance status (KPS)	28 (70 %)	6 (43 %)	6 (75 %)	21 (57 %)	
<70 ≥70	12 (30 %)	8 (57 %)	2 (25 %)	16 (43 %)	0.245
Lung metastases	13 (33 %)	6 (43 %)	4 (50 %)	15 (41 %)	0.748
Liver metastases	7 (18 %)	1 (7 %)	4 (50 %)	15 (41 %)	0.017
Bone metastases	13 (33 %)	6 (43 %)	4 (50 %)	25 (68 %)	0.020
Locoregional recurrence	13 (33 %)	9 (64 %)	4 (50 %)	11 (30 %)	0.104
Brain metastases (parenchymal)	11 (28 %)	12 (86 %)	4 (50 %)	13 (35 %)	0.001
Three methods of treatment used^b^	12 (30 %)	2 (14 %)	1 (13 %)	9 (24 %)	0.452

^a^In 21 patients with locally advanced or disseminated breast cancer, the diagnosis was established by fine needle biopsy before systemic therapy

^b^Intrathecal treatment and radiotherapy and systemic treatment delivered sequentially

Median time from the initial diagnosis of breast cancer to dissemination of the disease to any organ (disease-free survival DFS) was 12 months. Median survival from the initial diagnosis of breast cancer to LM was 25 months. Median survival from the detection of LM to death was 18 weeks (4.2 months, range 1–37 months). Six-month survival was 30 %, 1-year survival was 16 % and 2-year survival was 7 %. Median overall survival (OS) calculated from the initial diagnosis of breast cancer to death was 34 months. There were no statistically significant differences in survivals between biological subtypes of breast cancer. Survivals are presented in Table 4. Median survival of patients with good performance status (KPS ≥ 70) was 7 months and in those with poor performance status (KPS < 70) was 3 months (*p* < 0.001). Median survival of patients in whom systemic treatment was used was 6 months and in those without systemic treatment was 2 months (*p* < 0.001).

**Table 4:** Survivals of the entire group (118 patients) and within biological subsets

Median survival	Months	95 % CI	*p* value
*Disease-free survival* (*from the initial diagnosis of breast cancer to dissemination of the disease*—*any organ*)			
Entire group	12	8.604;14.664	
Triple-negative	14	6.756; 21.324	
HER2	10	5.388; 13.944	
Luminal B	10	0.000; 40.092	
Luminal A	16	5.964; 26.196	0.736
*Survival from the initial diagnosis of breast cancer to LM*			
Entire group	25	17.100; 33.600	
Triple-negative	24	15.336; 31.740	
HER2	12	0.000; 27.780	
Luminal B	39	0.000; 87.96	
Luminal A	32	20.136; 43.176	0.644
*Survival from LM the detection to death*			
Entire group	4.2	3.360; 5.196	
Triple-negative	3.2	1.572; 4.740	
HER2	4.6	1.968; 7.296	
Luminal B	3.4	0.000; 7.248	
Luminal A	4.2	2.436; 5.976	0.482
*Overall survival* (*from the initial diagnosis of breast cancer to death*)			
Entire group	34	[28.128;40.452]	
Triple-negative	32	[20.988;43.320]	
HER2	28	[14.388;41.316]	
Luminal B	41	[0.000; 87.756]	
Luminal A	36	[29.244;43.152]	0.457

Univariate analysis revealed that factors influencing survival from the detection of LM were bone metastases associated with LM, KPS, systemic therapy and radiotherapy to the brain. Multivariate analysis showed that out of 13 analysed variables, only KPS and systemic treatment (intravenous/oral chemotherapy, hormonal therapy, targeted therapy) were factors influencing survival from LM. The results of univariate and multivariate analysis are presented in Table 5. The analysis of the cause of death revealed that 82 % of patients died because of CNS progression, 8 %—due to progression in viscera (lungs or liver) and 10 %—due to progression in many organs (extra- and intracranial progression).

**Table 5:** Factors influencing survival from detection of LM—univariate and multivariate analysis

Factor	Univariate analysis	Multivariate analysis
*Karnofsky performance status* (*KPS*)		
≥70 vs. <70	*p* < 0.001	*p* = 0.015; HR = 0.485
*Biological subtype*		
Triple-negative vs. HER2-positive	*p* = 0.868	*p* = 0.782
Triple-negative vs. luminal A	*p* = 0.258	*p* = 0.290
*Histological type*		
Lobular vs. ductal carcinoma	*p* = 0.664	*p* = 0.485
Age <50 vs. >50	*p* = 0.575	*p* = 0.452
*Extracranial disease present*	*p* = 0.499	*p* = 0.238
*Localization of metastases*		
Lung	*p* = 0.886	*p* = 0.972
Liver	*p* = 0.613	*p* = 0.117
Brain (parenchyma)	*p* = 0.807	*p* = 0.704
Bones	*p* = 0.032	*p* = 0.819
*Type of treatment*		
Radiotherapy of the spinal cord	*p* = 0.259	*p* = 0.894
Radiotherapy of the brain	*p* = 0.017	*p* = 0.817
Intra-CSF chemotherapy	*p* = 0.978	*p* = 0.139
*Systemic therapy* (chth, ht, targeted)^a^	*p* < 0.001	*p* = 0.012; HR = 0.477
Intensity of treatment used		
3 methods vs. less	*p* = 0.119	*p* = 0.220

^a^chth-intravenous/oral chemotherapy; ht-hormonal therapy; targeted therapy

## Discussion

### Propensity of histological types and biological subtypes for leptomeninges

In the present study, a significant over-representation of lobular histologic type as well as triple-negative subtype in patients with LM was found.

A predisposition of lobular histological type to leptomeninges was confirmed by other authors, and this may be due to changes in cell adhesion molecules [1, 6]. In the literature, the rate of lobular cancer among all consecutive patients with initial diagnosis of breast cancer was 17–28 % [7–9], and in our institution, it was 18 % (data not shown). In the present study, 35 % of patients with LM had lobular cancer (two times more than in all consecutive breast cancer patients). Contrary to this, in a cohort of 420 patients with parenchymal brain metastases, described in one of our previous papers, the rate of patients with lobular cancer was only 7 % [5] (2 times less than in all breast cancer patients). These results suggest different propensity of lobular cancer for brain parenchyma and for leptomeninges.

In the present study, the proportion of patients with LM in triple-negative, luminal A and HER2-positive (HER2 and luminal B) subtypes was 40.5, 37.5 and 22 %, respectively. In the literature, 21–37 % [7, 8, 10] of patients with LM was triple-negative. HER2-positive subtypes (HER2 and luminal B), which play a major role in dissemination to the brain parenchyma [5], involve leptomeninges less frequently, in the literature the rate was 10 % [7], 23 % [8] and 28 % [10].

Our results suggest that triple-negative (40.5 %) and luminal A (37.5 %) subtypes were the most frequent biological subtypes affecting leptomeninges, but it does not necessarily indicate that both have a special biological predisposition to metastasize to the leptomeninges.

**Fig. 1:**
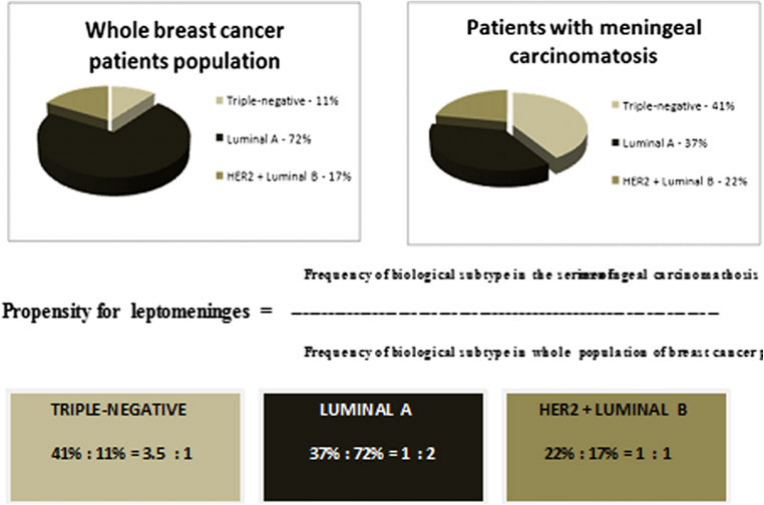
Propensity of biological subtypes for leptomeninges

To better analyse this problem, we had to find out the frequency of different breast carcinoma subtypes in whole population of breast carcinoma patients, so we assessed the rate of particular biological subtypes in 2,467 consecutive breast cancer patients treated in our institution in the years 2005–2006. The proportion of patients with luminal A, HER2-positive (luminal B and HER2) and triple-negative subtypes in whole population of newly diagnosed breast cancer patients was 72, 17 and 11 %, respectively. In the present study, the proportion of patients with breast cancer carcinomatous meningitis is 37.5, 22 and 40.5 %, respectively. Based on this data, the rate of luminal A, HER2-positive (luminal B and HER2) and triple-negative subtypes is about 2:1, 1:1 and 1: 3.5, respectively. The results suggest much higher propensity of triple-negative but not luminal A subtype to develop LM. The results are presented in Fig. 1.

The pattern of dissemination of breast cancer within biological subsets was comparable to our previous [11] and to other studies as well [12, 13].

### Survivals

Despite the intensive therapy, short median survival of only few months was described. In the present series, survival of patients with carcinomatous meningitis is slightly better (18 weeks) than observed in our previous study (16 weeks) [3], but it is still unsatisfactory. Median survival from LM is better than observed in the study done by Boogerd et al. [14] (12 weeks) and by Fizazi et al. [15] (14 weeks), it is comparable to the study conducted by Gauthier et al. [7] (17 weeks) and de Azevedo (3.3 months) [16] and is worse than in the study carried out by Wasserstrom et al. [17] (5.8 months). The 1-year survival rate was 16 %. It was higher than in the study done by Boogerd et al. [14] (11 %) and similar to the study performed by Wasserstrom et al. [17] (15 %), but lower in the study carried out by Gauthier et al. [7] (25 %) and de Azevedo [16] (24 %).

In the present study, we did not observe any difference in survival within biological subsets. Our data were comparable with the results by Lee et al. [10]. Surprisingly, when we analysed survivals of patients with parenchymal brain metastases [11], we observed differences in survival from the detection of brain lesions within particular biological subtypes. These results indicated that, there is a difference in the course of parenchymal brain and leptomeningeal metastases and confirmed the most deleterious character of LM.

### Factors influencing survival from LM

Out of 13 variables, only two factors influenced survival of patients with LM. They were performance status and systemic treatment. The role of performance status was confirmed in many studies [3, 6, 7, 14, 16, 18, 19], and its role in breast cancer patients with central nervous system involvement has been established.

During the last decade, the role of intravenous systemic treatment in patients with solid tumours and CM was postponed but recently, its role was established in several studies [2, 19–25]. Thick leptomeningeal metastases are well vascularized and thus could be better penetrated by systemically administered drugs than by intra-CSF agents, which penetrate only 2–3 mm into such lesions. Phase II studies with methotrexate [23], temozolomide [26] and topotecan with ifosfamide [27] in breast cancer patients with LM revealed 10–81 % response rate. In case reports using systemic drugs in patients with breast cancer leptomeningeal metastases radiological response and clinical improvement after capecitabine [28, 29], hormonal therapy [30] and trastuzumab [31] were shown. The older studies also confirm the role of systemic treatment in LM [14, 23, 25]. The data suggest that systemic treatment, especially new molecular drugs, used against both leptomeningeal and visceral metastases, could be more effective than only intrathecal treatment in patients with responsive systemic disease, especially in cases with nodular LM [19, 32]. In the present study, we confirmed the chemosensitivity and effectiveness of oral and intravenous chemotherapy, hormonal therapy and targeted therapy on patients with breast cancer LM.

## Conclusions

Out of four biological subtypes of breast cancer triple-negative subtype has the highest propensity to metastasize to the leptomeninges. Lobular histological type is also over-represented within patients with LM.

Performance status and systemic intravenous/oral therapy (chemotherapy, hormonal therapy and targeted therapy) are main factors determining survival of patients with LM.
